# Cracked mercury dental amalgam as a possible cause of fever of unknown origin: a case report

**DOI:** 10.1186/1752-1947-2-72

**Published:** 2008-03-06

**Authors:** Fabrizia Bamonti, Gianpaolo Guzzi, Maria Elena Ferrero

**Affiliations:** 1 Department of Medical Sciences, University of Milan, IRCCS Foundation Policlinico, Mangiagalli, Regina Elena Hospital, Via F. Sforza, 35, Milan, Italy; 2 Italian Association for Metals and Biocompatibility Research – A.I.R.M.E.B. Milan, Italy; 3 Institute of General Pathology, University of Milan, Via Mangiagalli 31, 20133 Milan, Italy

## Abstract

**Introduction:**

Sudden fever of unknown origin is quite a common emergency and may lead to hospitalization. A rise in body temperature can be caused by infectious diseases and by other types of medical condition. This case report is of a woman who had fever at night for several days and other clinical signs which were likely related to cracked dental mercury amalgam.

**Case presentation:**

A healthy women developed fever many days after had cracked a mercury dental amalgam filling. Blood tests evidenced increased erythrocyte sedimentation rate, anemia and elevated white cell count; symptoms were headache and palpitations. Blood tests and symptoms normalized within three weeks of removal of the dental amalgam.

**Conclusion:**

This case highlights the possible link between mercury vapor exposure from cracked dental amalgam and early activation of the immune system leading to fever of unknown origin.

## Introduction

There is enough evidence to suggest that mercury vapor and dental amalgam can be highly toxic[1]. Dental amalgam is the main source of mercury body burden. Mercury from maternal amalgam fillings has been shown to lead to a significant increase in mercury levels in the tissues and hair of fetuses and newborn infants [1]. In this case cracked mercury dental amalgam appears to be correlated with the symptoms experienced by our patient.

## Case presentation

**Figure 1:**
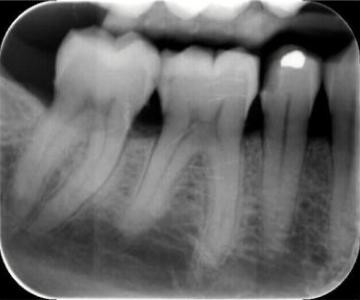
Endo-oral X-ray of the fractured dental amalgam.

In March 2007, a healthy 63-year-old woman presented to our dental center because of a broken mercury amalgam filling. During the previous two to three days she had experienced a slight rise in temperature at night, of apparently unknown origin. Four weeks prior to presentation, during routine oral hygiene with dental floss, she had cracked a ten-year old mercury dental amalgam filling, the only one in her mouth, located in the mandibular right second premolar. Examination revealed the presence of fractured occlusal surface dental amalgam leaving a partially empty cavity in the tooth. A dental X-ray showed no evidence of inflammation or infection (see Figure 1). The patient did not smoke or drink alcohol, had never been occupationally or environmentally exposed to mercury or other heavy metals, and only ate fish once a month. Interestingly, she constantly chewed gum, masticating about two pieces of chewing gum per day for six or seven hours running.

We have previously observed a potential correlation between fever of unknown origin and mercury dental amalgam in people with high susceptibility to mercury, possibly related to genetic polymorphism[2], and so recommended removal of the remaining amalgam. In order to do so we followed our standard safe procedure [3] and were able to reduce room mercury vapour levels by 10^-4 ^(from 0.5–0.7 mg/m^3 ^to 0.00025–0.00045 during cutting) compared to the other previously used techniques.

The following day, blood tests were performed to evaluate her condition. The results showed the patient had a high erythrocyte sedimentation rate (66 mm/h), low hemoglobin concentration (11.4 g/dL), low hematocrit (34.4%) and an elevated white cell count (9.9 per cubic millimeter) with 10.8 percent lymphocytes and 80.1 percent neutrophilic granulocytes. Her symptoms worsened as she reported having a temperature: mild (37–37.5°C) during the day but higher (38.0–38.5°C) at night, palpitations, headache and sporadic chest pain on the left side. Two days later she developed a high temperature (39.1°C) which lasted day and night for three whole days and which was associated with palpitations, a severe headache and chest pain on the left side, and which did not respond to standard antipyretic therapies, which, in fact, seemed to make things worse. We recommended no pharmacological treatment, but a diet including plenty of water, tropical fruits, meat and vegetables and avoidance of seafood [4]. She had been drinking more than two liters of water, eating about 150 g of beef and two to three portions each of fruit and vegetables per day, and, in addition to this, she had been taking encapsulated fruit and vegetable juice powder supplementation for about six weeks.

In order to examine potential exposure to inhaled mercury vapor and subsequent systemic toxicity, we determined the levels of total mercury in blood, urine, and scalp hair by using atomic absorption spectrometry for blood and urine and inductively coupled plasma for scalp hair. Her levels of total mercury in the biological matrices were within the normal range (blood total mercury: 2.3 microg/L, cutoff <2.0; urine total mercury 0.3 microg/L, cutoff<1.4; scalp hair total mercury 0.69 microg/g, cutoff<1.1 microg/g). These results indicate that the size of the remaining amalgam surface – accounting for 6 mm square (Figure 1) – was not big enough to increase mercury levels in the blood and urine in our patient. Moreover, the detected low levels of mercury in her scalp hair indicate that mercury vapor was the source rather than other species of mercury (methyl- and ethyl-mercury).

Idiosyncratic non-allergic toxic reactions to mercury may be independent of the exposure dose [2]. There was no evidence of any other symptoms connected with mercury toxicity, such as gingivitis, tremors, paresthesia, and tunnel vision [5]. Despite elevated concentrations of serum soluble interleukin-2 receptor, indicating an early immune activation, we decided not to perform this assay [2]. However the increase in erythrocyte sedimentation rate value was itself an indicator of immune activation. In fact its increase was related to the acute phase protein production by the liver, due to stimulation by cytokines released from activated immune cells.

Exposure to mercury vapor leaking from the cracked amalgam surface lasted four weeks. Mercury vapor is constantly emitted from amalgam surfaces and its release increases considerably during mastication, due to wear-abrasion. Prolonged exposure to chewing gum causes a sharp rise in intra-oral mercury vapor level. We believe that a higher level of mercury vapor is released from cracked amalgam than from a previously intact amalgam filling, particularly during gum-chewing.

With regard to idiosyncratic immunotoxic reactions, we believe that mercury vapor may cause systemic adverse events, independent of the dose.

Erythrocyte sedimentation rate value (18 mm/h), hemoglobin (12.2 g/dL), hematocrit (36.2%) and white-cell count (5.3 per cubic millimeter), as well as lymphocyte and neutrophil percent values, normalized within three weeks of removal of the dental amalgam and the patient's symptoms resolved.

## Discussion

This case suggests that cracked dental mercury amalgam can be considered a possible cause of fever and other clinical symptoms. There is plenty of evidence to suggest that mercury and its chemical compounds have quite a high level of toxicity and particularly dental mercury amalgam which was one of the most commonly used materials for dental restoration [6,7].

The elevated white cell count observed in this patient was not related to a viral infection because of the lack of percent lymphocyte increase, which, on the contrary, was much lower than normal. It has been previously reported that mercury released from dental silver fillings increases the incidence of mercury- and antibiotic-resistant bacteria in the oral and intestinal flora of primates [8]. Even if rejected at first because of lack of response to standard antipyretic therapies, the hypothesis of mercury-resistant bacterial enrichment in normal floras cannot be ruled out considering that this patient's fever returned to normal three weeks after removal of the mercury amalgam. Finally, low haemoglobin concentration and low hematocrit were related to anemia, which was possibly provoked by the toxic effect of mercury on bone marrow erythropoiesis.

In our opinion, early recognition and removal of sources of mercury, together with improved diet and vitamin supplementation, can prevent damage to the immune system.

## Conclusion

This case suggests that it is worth investigating whether a fever of unknown origin is due to exposure to a source of mercury.

## Competing interests

The author(s) declare that they have no competing interests.

## Authors' contributions

FB collected the biochemical and clinical data. GG performed the endo-oral X-ray and managed the patient. MEF had the original idea and wrote the paper. All authors have read and approved the final manuscript.

## Consent

Written informed patient consent was obtained from the patient for publication of this case report and the accompanying image. A copy of the written consent is available for review by the Editor-in Chief of this journal.
